# Examining the Implementation of Conditional Financial Incentives Using the Reach, Effectiveness, Adoption, Implementation, and Maintenance (RE-AIM) Framework to Improve HIV Outcomes among Persons Living with HIV (PLWH) in Louisiana

**DOI:** 10.3390/ijerph19159486

**Published:** 2022-08-02

**Authors:** Sarah Chrestman, Tejal Patel, Katherine Lass, Catherine Maulsby, Hayley Alexander, Charlie Schwanz, Kimberley O’Brien, Waref Azmeh, Austin Matthews, Latoya Decuir, Dionne Bell, Julie Cacioppo, Tina Martinez, Julie D. Doyle, Angie J. Brown, Shamekia Wave, Rubina Abrol, Tammeka Evans, Russell Brewer

**Affiliations:** 1 Louisiana Public Health Institute, New Orleans, LA 70130, USA; schrestman@lphi.org (S.C.); schwanz.cz@gmail.com (C.S.); 2 School of Public Health and Tropical Medicine, Tulane University, New Orleans, LA 70112, USA; tpatel2@tulane.edu; 3 The Policy & Research Group (PRG), New Orleans, LA 70118, USA; katie@policyandresearch.com; 4 Bloomberg School of Public Health, Johns Hopkins University, Baltimore, MD 21205, USA; cathymaulsby@gmail.com; 5 Louisiana Department of Health, Bureau of Planning and Performance, Baton Rouge, LA 70821, USA; hayley.alexander@la.gov; 6 AHF Baton Rouge Healthcare Centers, Baton Rouge, LA 70809, USA; kimberley.obrien@ahf.org (K.O.); waref.azmeh@aidshealth.org (W.A.); 7 Social Research and Evaluation Center, Louisiana State University, Baton Rouge, LA 70803, USA; amatthews2@lsu.edu; 8 CareSouth Medical and Dental, Baton Rouge, LA 70806, USA; ldecuir@caresouth.org (L.D.); dbell@caresouth.org (D.B.); 9 Our Lady of the Lake Regional Medical Center, Baton Rouge, LA 70808, USA; julie.cacioppo@fmolhs.org (J.C.); tina.martinez@fmolhs.org (T.M.); julie.doyle@fmolhs.org (J.D.D.); angelee.brown@fmolhs.org (A.J.B.); 10 Multispecialty Care Clinic, Baton Rouge, LA 70806, USA; sjohnson@drgertie.com; 11 The Medical Affairs Company, Kennesaw, GA 30144, USA; rabrol1220@hotmail.com; 12 ViiV Healthcare, Research Triangle Park, Durham, NC 27709, USA; tammeka.m.evans@viivhealthcare.com; 13 Department of Medicine, University of Chicago, Chicago, IL 60637, USA

**Keywords:** financial incentives, HIV care continuum, South, people living with HIV, implementation science, RE-AIM

## Abstract

Economic strengthening interventions are needed to support HIV outcomes among persons living with HIV (PLWH). The Baton Rouge Positive Pathway Study (BRPPS), a mixed method implementation science study, was conducted to assess key RE-AIM components tied to the provision of conditional financial incentives among PLWH in Baton Rouge, Louisiana. Seven hundred and eighty-one (781) PLWH enrolled at four HIV clinic sites were included in the final analyses. Participants completed an initial baseline survey, viral load test, and were contacted at 6 and 12 months (±1 month) post-enrollment for follow-up labs to monitor viral load levels. Participants received up to USD140 in conditional financial incentives. The primary analyses assessed whether participation in the BRPPS was associated with an increase in the proportion of participants who were: (a) engaged in care, (b) retained in care and (c) virally suppressed at baseline to 6 and 12 months post-baseline. We constructed a longitudinal regression model where participant-level outcomes at times t_0_ (baseline) and t_1_ (6- or 12-month follow-up) were modeled as a function of time. A secondary analysis was conducted using single-level regression to examine which baseline characteristics were associated with the outcomes of interest at 12-month follow-up. Cost analyses were also conducted with three of the participating clinics. Most participants identified as Black/African American (89%). Fewer than half of participants reported that they were unemployed or made less than USD5000 annually (43%). Over time, the proportion of participants engaged in care and retained in care significantly increased (70% to 93% and 32% to 64%, *p* < 0.00). However, the proportion of virally suppressed participants decreased over time (59% to 34%, *p* < 0.00). Implementation costs across the three sites ranged from USD17,198.05 to USD396,910.00 and were associated with between 0.37 and 1.34 HIV transmissions averted at each site. Study findings provide promising evidence to suggest that conditional financial incentives could help support engagement and retention in HIV care for a high need and at risk for falling out of HIV care population.

## 1. Introduction

Within the last decade, there has been increased attention on improving HIV continuum of care (HCC) outcomes among persons living with HIV (PLWH) given its association with improved morbidity and mortality as well as reductions in new HIV infections at a population level [1,2,3]. A range of HIV care and treatment targets have been described in several HIV response initiatives including the UNAIDS Fast Track cities, the National HIV/AIDS Strategy, and the more recent Ending the HIV Epidemic Plan [4,5,6]. Jurisdictions across the country are charged with developing community-engagement plans and implementing evidenced-based and/or promising interventions in order to improve HCC outcomes within highly HIV-impacted jurisdictions [7,8,9,10]. Implementation research is a useful approach that can be utilized to better understand the strengths and limitations of implementation strategies that are being used to improve access to HIV services, how they can be improved in real time, their mechanism of change, and within which context they operate to improve HCC outcomes for PLWH [11,12].

The utilization of economic strengthening approaches (e.g., financial incentives) within HIV clinic settings may serve as a useful intervention strategy to improve HCC outcomes for PLWH [13,14,15]. Financial incentives, in general, have been shown to be effective in achieving desirable health behaviors and positive effects [16,17]. However, the specific use of financial incentives to improve HCC outcomes (e.g., engagement in HIV care, HIV medication adherence, and viral suppression) among PLWH has shown mixed findings [18,19,20,21,22,23,24]. There have also been limited studies among highly HIV-impacted Southern populations in the United States [18]. In addition, financial incentives may hold significant promise within specific contexts and for specific segments of PLWH (e.g., those not consistently virally suppressed) [19].

In 2018, the Louisiana Public Health Institute (LPHI) was awarded 3-year funding from ViiV Healthcare to implement and evaluate a conditional financial incentive study called the Baton Rouge Positive Pathway Study (BRPPS) within clinical settings. BRPPS, a mixed method implementation study, utilized the Reach, Effectiveness, Adoption, Implementation, and Maintenance (RE-AIM) framework with key implementation outcomes to guide the study [25,26,27]. RE-AIM is a useful framework that provides structure to health program impact evaluations [26]. It also focuses on contextual and setting factors, which aid in understanding which components work well [26]. The primary aim of BRPPS was to examine specific RE-AIM components tied to the implementation of conditional financial incentives among PLWH in the Baton Rouge area.

The specific strategy (i.e., the utilization of conditional financial incentives) was prioritized and selected for implementation during a broader community brainstorming meeting with Baton Rouge stakeholders prior to study implementation. The Baton Rouge area was selected because of its existing implementation partner infrastructure and interest as well as the high burden of HIV in this jurisdiction, particularly among Black/African American individuals who are underserved and highly marginalized [28,29].

The Baton Rouge area ranks high in HIV/AIDS case rates among large United States (US) cities and is emblematic of the disproportionate burden of HIV in the Southern US [28,29]. In 2016, there were 238 new HIV diagnoses and more than 5000 PLWH in the Baton Rouge area [30]. Community partners also perceived this project as an opportunity to improve the health of PLWH who were not receiving the full benefits of HIV services given that of all PLWH in the metropolitan area, 4005 (79%) were engaged in HIV care and an estimated 21% (1068) were not receiving the full benefits of HIV care and treatment [31]. The Baton Rouge area also supports access to HIV care and treatment for PLWH through a combination of private insurance, Medicaid, and Ryan White-funded services including the AIDS drug assistance program (ADAP) [32].

In summary, the current study builds on previous studies and knowledge with its focus in a highly HIV-impacted Southern jurisdiction as well as among a high need and at risk for falling out of HIV care population.

## 2. Methods

The study was approved by an external IRB (#18079) on 28 July 2018 and registered with Research Registry [33]. Verbal informed consent was obtained prior to enrollment. Participants completed a baseline (at enrollment) and follow-up survey at 12 months (±1 month) post enrollment. Participants were compensated USD50 in the form of a Visa gift card for the initial baseline survey and viral load test. They also received USD15 for the follow-up survey. In addition, conditional financial incentives in the form of a Visa gift card were distributed at each of the participating HIV clinic sites to enrolled PLWH based on the achievement of specific HCC milestones outlined in Table 1. We also specify and report on the implementation strategy [34] in Table 2. HIV clinic staff conducted the study assessments and administered the financial incentives.

In order to qualify for the study, potential participants had to: (a) have a confirmed HIV diagnosis based on review of medical records and/or provided documents; (b) self-report that they were living in the Baton Rouge area and did not plan to move within the next 12 months; (c) self-report that they were at least 18 years or older; (d) report that they were able to conduct the study in English and provide verbal consent; and (e) report at least one additional criterion from Category A or B.

Categories A and B were informed by the participating HIV clinics and included PLWH who were perceived as a high need population for HIV services and a population at high risk for falling out of HIV care. The category A (*n* ≥ 80% of sample) criteria consisted of PLWH who had not been in HIV care for 8 months or more (i.e., no HIV viral load test); or had two consecutive detectable viral load results ≥200 copies/mL at least 90 days apart; or had missed 2 or more visits in the last six months prior to enrollment. Category B (*n* ≤ 20% of sample) criteria consisted of PLWH who were newly diagnosed with HIV more than 3 months ago and had not accessed HIV care (i.e., no HIV viral load test); or who were at risk for falling out of care by self-reporting at least one risk factor: unstable housing, unemployment for 3 months or more, incarceration history, and/or a recent behavioral health condition (e.g., diagnosis and/or treatment history) within the 12 months prior to enrollment.

We aimed to enroll 1000 participants from HIV clinic sites in the Baton Rouge area. Diverse HIV clinics participated in the study including a non-profit health care center, federally qualified health center, hospital system, and two private practices. Site-specific enrollment targets were pre-determined based on each site’s capacity and service population. All enrolled PLWH received standard of care services such as health navigation, case management, and/or treatment adherence counseling at the participating HIV clinic sites. Participants were recruited using flyers, via word of mouth, from clinic walk-ins, via electronic medical records (EMR), and from existing out of care lists that were generated by each of the HIV clinic sites. Potential participants were excluded from the study if they were not willing or unable to provide informed consent, were currently participating in another study focused on improving HCC outcomes, and/or were currently institutionalized (i.e., hospitalized or imprisoned).

BRPPS’ evaluation was guided by the RE-AIM Framework and assessed all five RE-AIM dimensions: Reach, Effectiveness, Adoption, Implementation, and Maintenance [25,26]. We assessed seven key implementation outcomes including penetration, effectiveness, adoption, acceptability, feasibility, cost, and sustainability, which were aligned with the RE-AIM dimensions. They are described in Table 3. *Reach (penetration)* was defined as the number of enrolled participants among the estimated number of potentially eligible participants from the included clinics [25,26,27]. This dimension was assessed from the participating clinics. *The promise of effectiveness* [25,26,27] of conditional financial incentives was assessed using three primary outcomes: engagement in HIV care, retention in HIV care, and viral suppression. Engagement in HIV care was defined as the proportion of established participants who have at least one viral load test within a 12-month period (±1 month) since enrollment [35]. Retention in HIV care was defined as the proportion of participants who have at least 2 viral load tests at least 90 days apart within a 12-month period (±1 month) since enrollment [35]. The baseline visit was not included in the determination of the retention and engagement outcomes at baseline. Viral suppression was defined as the proportion of participants who achieve a viral load result of <200 copies/mL at each reporting period (6 and 12 months post-enrollment) [35]. The predicted probabilities of achieving the three HCC outcomes were obtained using the margins post-estimation command in Stata 15 [36].

*Adoption* was assessed at the setting level [25,26,27] and defined as follows: (1) the number of HIV clinic sites that agreed to participate in the study; (2) the number of HIV clinic sites that actually completed data collection activities and distributed incentives; and (3) the percentage of participants that were virally suppressed who received their financial incentives at 6- and 12-month follow-up which was monitored using incentive tracking forms.

*Individual-level acceptability* [25,26,27] was assessed in the baseline survey and examined the extent to which participants agreed or disagreed with the following four statements: financial incentives can improve overall health, financial incentives can improve viral load numbers, financial incentives can improve clinic attendance, and financial incentives should not be provided to persons living with HIV as it is something they should already be doing. A mean acceptability score was calculated by taking the average of responses to all four items with a range of 0 (strongly disagree) to 4 (strongly agree). Prior to calculating the acceptability score, the fourth item was first reversed scored. Only participants who responded to all four questions were assigned a mean acceptability score and included in the analysis (complete case analysis).

*Ongoing barriers to implementation* [25,26,27] were qualitatively assessed and discussed during ongoing calls with the participating clinics. *Feasibility* [25,26,27] was defined as the extent to which all HIV clinic sites achieved their planned and/or revised enrollment targets. *Cost, cost utility and threshold analyses* were assessed using an adapted micro-costing Excel worksheet which had been utilized to conduct economic evaluations of HIV prevention, linkage, and retention in care programs [37,38]. A consultant worked closely with the research team to collect the planned cost data. Participating HIV clinic sites collected cost data from their accounting records with brief telephonic support provided by the research team to estimate the cost of program implementation from the societal and payer perspectives.

*Maintenance (sustainability)* was examined at the setting level [25,26,27]. Maintenance was operationalized as the extent to which a program or policy becomes institutionalized or part of the routine organizational practices and policies [25,26,27]. This construct was qualitatively assessed on the ongoing calls with the HIV clinic sites.

## 3. Data Analysis

Descriptive analysis was conducted to describe the sample of participants. We examined variable distributions and identified outliers and inconsistent values. Measures of central tendency (e.g., means, medians, etc.) and frequencies to characterize the study population. The primary design used to examine the promise of effectiveness was a treatment-only investigation of change in HCC outcomes from pre- to post-enrollment points in time. We assessed the extent to which participants were more or less likely to be: (a) engaged in care, (b) retained in care, or (c) virally suppressed over time from baseline (pre-enrollment) to follow up (6 and 12 months post-enrollment). For each of the three HCC outcomes of interest (engagement, retention, suppression), we constructed a longitudinal regression model where participant-level outcomes were modeled as a function of time, with time operationalized as a continuous counter variable whose values range from 0 to 2.

A secondary analysis was conducted to assess the baseline correlates of engagement in care, retention in care, and viral suppression at 12-month follow-up. For each outcome of interest, we construct a series of single-level regression models, where HIV care outcomes observed at the 12-month follow-up were regressed on a set of participant-level characteristics observed at baseline, the baseline HIV care outcome measure, and clinic/site controls. Independent variables included in the benchmark model were age at enrollment, race/ethnicity, gender, relationship status, employment status, housing situation, insurance status, site of enrollment, and enrollment category. Additional analyses were conducted for each of the remaining, but comparatively incomplete, independent variables of interest (i.e., sexual orientation, education, income, incarceration history, years living with HIV, depressive symptoms, medication adherence, alcohol use, internalized HIV stigma, and incentive acceptability) in which the benchmark set of predictors was also included in the model as controls, and only the relationship between the additional variable and outcome is reported. Coefficients and their corresponding *p*-values were used to identify which characteristics were most associated with the HCC outcomes at 12-month follow-up while controlling for enrollment site and the baseline HCC outcome measures.

*Cost, Cost Threshold and Utility Analyses*. Three clinical sites participated in the cost analyses. The first three sites completed most of the cost data collection prior to March 2020. Having joined late, the fourth site had not collected cost data and opted out of the cost analyses due to pandemic constraints. The consultant utilized standard cost analytical methods recommended by the United States Panel on Cost- Effectiveness in Health and Medicine [39]. The first step consisted of a cost analysis to assess the actual resources consumed by the program, rather than estimated or planned costs. The overall cost of the conditional financial incentive intervention was calculated by summing all resources consumed during the timeframe for the analysis. The consultant computed the cost per client by dividing the total costs of the intervention by the total number of participants. Evaluation costs were excluded from the cost analysis. A threshold analysis was also conducted to determine the number of HIV infections that would need to be averted to make the claim that the intervention was cost saving. This threshold was computed as [C/(T + (Q * W))]. In this formula, C = the total cost of the program (calculated by summing the total cost of the three phases), T = lifetime medical costs averted for each HIV transmission averted; Q = Quality adjusted life years (QALYs) and W = price that society is willing to pay for a QALY. Based on previous studies, the lifetime cost of HIV medical care (T) will be USD382,954 and societal cost for a QALY (W) = USD195,838.58 [40,41]. QALYS were computed through C/W. Cost data were regionally indexed based on each clinical site’s location.

*Cost Utility Analyses*. To assess cost-effectiveness, the consultant calculated “R” the cost–utility ratio (or net cost per QALY) using the following formula: R = (C − AT)/AQ. “A”, the number of HIV infections averted by each program, was determined by multiplying the number of person-years of viral suppression achieved by the program by the expected change in the estimated annual transmission rate for an individual who is not virally suppressed vs. virally suppressed. We estimated person-years of viral suppression by calculating the net number of participants who become virally suppressed during the program. We used an annual transmission rate of 6.1% [42]. To estimate the total number of QALYs saved, we assessed QALYs saved through the improved health of program participants (Q1) by multiplying the estimated number of person-years of viral suppression by 0.039 [43], an estimate of the number of QALYs saved from improvements in quality of life [44]. To estimate the number of QALYs saved through averted HIV infections (Q2), we multiplied the estimate of HIV infections averted by the program (A) by 5.83, the estimate from the literature of the number of QALYs saved by an averted HIV infection [40]. We summed the total number of QALYs saved by adding Q1 to Q2. We consider programs with a cost per QALY of less than USD195,838 to be cost-effective [45].

## 4. Results

*Demographic characteristics.* Participant characteristics are described in Table 4. This represents data from four of the five participating HIV clinic sites as study activities were discontinued at one of the sites. The majority of participants (89%) identified as Black/African American. Very few participants identified as Hispanic or Latinx (2%). Roughly one-half of participants identified as male (53%), 45% as female (45%), and 2% as transgender or non-binary. Approximately two-thirds of participants identified as heterosexual (68%) and about one-third as gay, lesbian, bisexual, or other sexual minority category (32%). Twenty-two (22%) percent of participants were Black men who have sex with men (not reported in Table 4). Less than 20% of participants reported having a primary partner. Almost all participants reported having health insurance (90%). More than half of all participants reported earning less than USD10,000 a year (58%). Just over one-third (37%) reported being employed either full- or part-time/occasionally at intake and 14% reported unstable housing. In addition, 12% of participants reported a recent history of incarceration. Nearly all participants reported living with HIV for at least one year at baseline (91%), a small portion (9%) reported a more recent diagnosis (Table 4).

*Psychosocial and Behavioral characteristics.* These characteristics are reported in Table 4. Approximately one-third of cis-gender male participants reported hazardous alcohol use (i.e., ≥4 points), while 29% of cis-gender female participants reported hazardous alcohol use (i.e., ≥3 points) based on the AUDIT-C alcohol consumption scale [46]. Seven percent (7%) of gender minority participants reported hazardous alcohol use (i.e., ≥3 points) based on the AUDIT-C alcohol consumption scale using the recommended cutoff score of 3 to predict alcohol dependence symptoms or consequences for gender minority individuals [47]. Participants reported mild depressive symptoms based on the Patient Health Questionnaire (PHQ)-9 [48] at intake (7.2 out of 24). Participants self-reported good HIV medication adherence (score of 11.8 out of 16) based on the CASE Adherence Index [49] and high levels of internalized HIV stigma (score of 4.6 out of 5) based on an adapted internalized HIV stigma scale [50]. Most participants were engaged in care (70%) and virally suppressed (62%) at enrollment with less than half (42%) meeting the retained in care definition.

*Reach.* Participants were recruited from five HIV clinics in the Baton Rouge area. The study initially aimed to enroll 1000 participants but this target was reduced to 800 due to the late start date attributed to delays in obtaining initial IRB approval. Eight hundred and sixteen (816) of an estimated 1950 potentially eligible participants were enrolled in the BRPPS from August 2018 through October 2019. However, study activities were discontinued at one site due to challenges associated with retrieving study data, and information from 35 participants enrolled at that site were excluded from the final analyses. Thus, 781 enrolled participants from the remaining four sites were included in the final analyses.

**Figure 1:**
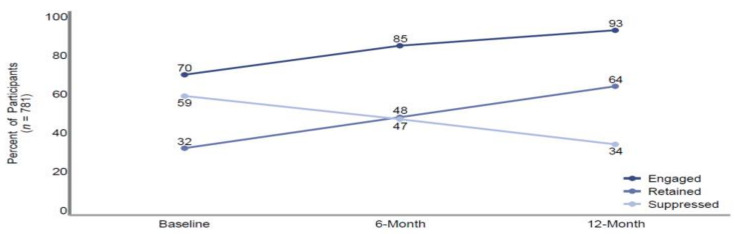
Predictive probability of achieving the three HIV care outcomes of interest over time, BRPPS, Baton Rouge, LA.

*Promise of Effectiveness.* The predicted probabilities of achieving the three HCC outcomes of interest over time are shown in Figure 1. Results from the longitudinal models suggest that the proportion of participants who engaged in HIV care significantly increased over the course of the study from 70% at baseline to 85% at 6-month and eventually to 93% at 12-month follow-up (r = 1.28, SE = 0.11, *p* < 0.00). Rates of retention in HIV care also significantly increased over the course of the study. At baseline, fewer than one-third (32%) of participants were retained in care with retention increasing to 48% at 6-month follow-up and then to 64% at 12-month follow-up (r = 0.75, SE = 0.06, *p* < 0.00). However, the proportion of participants who were virally suppressed decreased over time. At baseline, 59% of participants were virally suppressed. At 6-month follow-up, that number decreased to 47% and eventually to 34% at 12-month follow-up (r = −0.65, SE = 0.07, *p* < 0.00).

As shown in Table 5, after controlling for baseline HCC outcomes and site, we found few statistically significant relationships between the baseline covariates and HCC outcomes at 12-month follow-up. Age at intake was positively and significantly associated with two HCC outcomes (i.e., retention [*p* = 0.001] and viral suppression [*p* = 0.029]) at 12-month follow-up, suggesting that older participants were more likely to be retained in care or virally suppressed compared with younger participants. Enrollment site estimates showed that participants enrolled at Site D were significantly less likely to be engaged in HIV care at 12-month follow-up (r = –1.84 (0.72), *p* = 0.011). Participants with unstable housing were less likely to be engaged in care compared with participants with stable housing (*p* = 0.051) and those enrolled under Category A were less likely to be retained in care (*p* = 0.053) or virally suppressed (*p* = 0.005) compared with those enrolled under Category B. Persons diagnosed with HIV less than a year prior to enrollment were significantly more likely to be retained in care and virally suppressed (*p* = 0.049 and 0.011, respectively) compared with participants who had been living with HIV for 5 or more years.

Interestingly, health insurance status was not associated with any of the three HIV care outcomes at 12-month follow-up, although this may be an artifact of the majority of study participants having health insurance and attending clinics that offer services funded through Ryan White. Across race, ethnicity, and gender groups, none of these baseline characteristics were associated with the three HIV care outcomes. Participants with more severe depressive symptoms were more likely to be engaged in HIV care at 12-month follow-up (*p* = 0.051) compared with those who had milder depressive symptoms. In contrast, having unstable housing and a history of incarceration were negatively associated with being engaged in care (*p* = 0.051 and 0.012, respectively). Similarly, compared with participants with no history of incarceration at intake, those who reported spending any time in jail or prison were 4% less likely to be engaged in care.

*Adoption.* BRPPS initially partnered with four clinical sites to recruit study participants. One site was unable to meet study recruitment/data reporting requirements, and thus a new site was on boarded to help reach our revised target enrollment of 800. There were differences in how the study was implemented at the final four sites included in the analysis. For example, while one site had eight in-house research staff to recruit, track, and report on participant progress, the other three sites had only 1–3 staff members dedicated to BRPPS. In many cases, site coordinators were responsible for recruitment, monitoring, and reporting data to the BRPPS research team. In total, 1533 incentives were distributed for participants’ labs. Just under half of participants (44%) received a viral suppression incentive at 6-month follow-up and 45% received a viral suppression incentive at 12-month follow-up (incentive data reported in Table 1).

*Feasibility.* The participating HIV clinic sites were able to exceed our revised target enrollment of 800. Eight hundred and sixteen (816) participants were enrolled in the BRPPS, thus recruitment was feasible and represents about 16% of all PLWH in the Baton Rouge area.

*Financial Incentive Acceptability.* Participants reported mild acceptability (Mean = 2.5) of financial incentives at baseline (Table 4). We did not observe a statistically significant relationship between financial incentive acceptability at baseline and any of the three HIV care outcomes at 12-month follow-up (Table 5).

*Barriers to implementation.* Participating HIV clinic staff alluded to the significant amount of time and effort that was required to manage and track incentive distribution on the ongoing monitoring calls. While participant recruitment was relatively easy for the HIV clinic sites to integrate during regular clinic intake appointments, sites often delayed in data reporting requirements, making it difficult to consistently monitor overall program implementation.

*Cost, Cost Utility and Threshold Analyses.* Finally, cost–utility and cost–threshold analyses demonstrated that financial incentives are a cost-efficient and feasible method for reducing HIV transmissions. Annual cost utility costs were calculated for January 2019–December 2019. Implementation costs across the three sites ranged from USD17,198.05 to USD396,910.00 and were associated with between 0.37 and 1.34 HIV transmissions averted at each site (Table 6). Thus, Site A averted at least one new HIV transmission compared with the other two sites included in the analysis. Site A also had a significantly higher implementation budget and site implementation budgets were based on projected enrollment numbers. For example, sites with higher enrollment numbers (e.g., site A) received greater funds for implementation. Total implementation costs for the BRPPS across all sites was USD438,449.09. Implementation of the intervention was considered to be cost-effective across all three sites as they were under the threshold of USD195,838.587 per QALY saved. Clinic A was considered to be a highly cost-effective site, with a cost of USD11,000 per QALY saved. Even when using the commonly conservative threshold of USD100,000, the other two sites were still considered to be cost-effective.

The cost-saving threshold, or the number of HIV transmissions that must be averted to deem each site cost-saving, ranged from 0.04 to 1.04 over a one-year period (Table 7). In other words, one or fewer HIV transmissions must be avoided for the program to be considered cost-saving. The cost-effectiveness threshold, or the number of QALYs that must be saved to deem the program cost-effective, ranged from 0.09 to 2.03 over a one-year period. Essentially, fewer than one to three QALYs must be saved for the program to be considered cost-effective.

### Maintenance

Participating HIV clinic staff expressed concern around the sustainability of incentives upon completion of the study on regular calls, especially if incentives had to come out of their operating budgets.

## 5. Discussion

The BRPPS was the largest conditional financial incentive study among PLWH focused entirely in the Baton Rouge area. Study findings provide promising evidence to suggest that conditional financial incentives may help support engagement and retention in HIV care but not viral suppression. Thus, financial incentives (i.e., up to USD140 for labs and viral suppression) may be a promising intervention strategy to engage and retain PLWH in HIV care but not necessarily in motivating medication adherence for viral suppression and/or addressing key structural barriers to medication adherence.

It is important to note study characteristics that mirrored the broader Baton Rouge HIV epidemic. Namely gender (majority male) and racial composition (majority Black/African American) of the current study were aligned with the broader Baton Rouge HIV epidemic [30]. Additional considerations include gender identity, sexual orientation, and unemployment/income levels. Transgender individuals comprised 2% of the BRPPS enrollees compared with the percentage of transgender individuals living with HIV (i.e., 1%) in the Baton Rouge area [30]. We also enrolled a significantly large percentage of heterosexual participants (i.e., 68%) in the study compared with the percentage of heterosexual PLWH (i.e., 31%) in the Baton Rouge area [30]. Unemployment estimates (43%) were higher among study participants compared with the state’s estimate among PLWH (25%) [51]. Income levels were particularly low among study participants compared with the state’s estimate where approximately 43% of study participants made less than USD5000/year compared with an estimated 6% of PLWH in the state who made less than USD5000/year [51]. Thus, participants enrolled in the BRPPS represented a low-income sample.

Our study findings are contrary to a previous 2013–2016 financial incentives study in Louisiana which enrolled more than 2000 PLWH across three urban HIV specialty clinics in Louisiana [17]. After 12 months of enrollment, the percentage of PLWH who were virally suppressed increased from 57.8% to 82.7% (*p* < 0.001) [17]. In addition, every subgroup experienced a substantial increase in viral suppression from baseline to 12 months follow-up except PLWH who were already virally suppressed at baseline [17]. Key differences between the two studies include: BRPPS utilized more restrictive criteria tied to level of HIV engagement and likelihood of falling out of care; BRPPS utilized Visa cards whereas the other study utilized cash payments on reloadable debit cards; BRPPS financial incentives were capped at USD140 whereas participants in the other study could earn additional incentives for attending medical appointments, laboratory appointments, and for viral suppression. This points to several explanations for our findings: (1) the incentive amount may have been too low; (2) participants may have preferred other forms of payment (e.g., cash); and (3) participants enrolled in BRPPS represent a high need population with substantial structural barriers.

HIV clinic sites were engaged in the planning phase of the study. During initial discussions with the HIV clinic sites, they indicated a preference for lower incentive amounts as larger incentive amounts were perceived as not being sustainable without a long-term funding source. Thus, the BRPPS utilized a smaller incentive amount (up to USD140 for the intervention component) based on initial partner recommendations in order to increase the likelihood of sustainability upon completion of the study. Additionally, the clinics requested the expansion of the inclusion criteria to include PLWH who were currently engaged in care but were considered to be at risk of falling out of care due to unstable housing, unemployment, incarceration history, and/or behavioral health conditions. Based upon their clinical experiences, they believed that this at-risk group would greatly benefit from conditional financial incentives. As a result, there was a large number of participants who were virally suppressed at baseline. Plans are underway to share the study results with the sites via a formal presentation and garner their input on the implications of the study and opportunities for dissemination.

In retrospect, an assessment of implementation and evaluation readiness prior to enrollment may have proved useful given that study activities at one of the sites were discontinued due to challenges associated with retrieving study data. The administration and tracking of financial incentives were also high burden activities. Additional strategies to reduce this burden would have been helpful.

Although we did not find a significant relationship between financial incentives and viral suppression, our cost analysis provided several insights for the field. First, the implementation of financial incentives was cost-effectives across the three sites that were included in the analysis. Second, the three sites included in the analysis represented clinics with 1–3 staff members that were involved in the study. Lastly, a limited number of studies have examined the impact of financial incentives from a cost perspective and cost is an alternative outcome to viral suppression that should be examined in future studies. A limitation of our cost analysis is that one site did not participate in the analysis.

In response to the developing COVID-19 pandemic, the Louisiana Department of Health mandated that all healthcare facilities in the state substantially restrict in-person client visits to those with the highest medical need beginning in March 2020. Some restrictions were lifted beginning in June of 2020; however, HIV clinic sites reported that staff continued to limit the scheduling of in-person clinic visits to only those who had acute needs or who could not be treated virtually through the fall of 2020. As a result, study coordinators reported that participants who had follow-up windows between March 2020 and the end of the study period (November 2020) experienced difficulties scheduling lab visit appointments during their appropriate windows. Thus, this may have also contributed to the unobserved relationship between conditional financial incentives and viral suppression.

There are additional limitations to report. A historical comparison was planned; however, this was not completed due to imbalanced samples and differences in how outcomes were constructed in the two studies. Our study was impacted by the COVID-19 pandemic. While enrollment had closed prior to the emergence of the COVID-19 pandemic, it affected participants’ ability to complete HIV labs due to stay at home orders and exacerbated barriers to care. As a result, participants’ completed labs fell outside of the original follow-up windows. The COVID-19 pandemic also influenced incentive distribution. At the end of the study, some participants earned incentives but had not yet received them. Many participants limited in-person visits during the pandemic, and their next in-person visit to pick-up the incentive was scheduled for a date after the close of the study. In those instances, the incentives were counted as distributed for analytic purposes since the participant would be receiving them at the next visit. Given the more stringent selection criteria, our findings are also limited to a high need and at risk for falling out of HIV care population. Lastly, our findings are not generalizable to all PLWH in the United States. However, it does represent a substantial percentage of all PLWH in the Baton Rouge area.

## 6. Conclusions

In conclusion, our findings can be used to inform Ending the HIV Epidemic efforts [8] in the Baton Rouge area. Financial incentives may be a promising intervention strategy to engage and retain PLWH in HIV. In this instance, they were also cost-effective for a high need and at risk for falling out of care Southern population. They may also need to be coupled with implementation strategies to reduce incentive administration and tracking burden as well as additional strategies focused on adherence and/or the social determinants of health given that specific subpopulations (i.e., persons with unstable housing, a history of incarceration, and younger individuals) were less likely to be linked, retained, and/or achieve viral suppression.

## Figures and Tables

**Table 1:** Description of Baton Rouge Positive Pathway Study (BRPPS) conditional financial incentives schedule for specific HCC-related activities and outcomes among enrolled persons living with HIV.

Incentivized Events	Incentive Type	Amount	Frequency of Incentive	Total Amount	Number Received
Completion of subsequent HIV laboratory testing up to 4 per year	Intervention	USD10	4	USD40	637 *
Achievement of viral suppression at 6-months post-enrollment	Intervention	USD50	1	USD50	343
Achievement or maintenance of viral suppression at 12-months post enrollment	Intervention	USD50	1	USD50	349

* 637 received at least 1 lab test incentive, 501 received at least 2, 288 received at least 3, and 107 received 4.

**Table 2:** Specification and reporting on the conditional financial incentive strategy for the BRPPS.

Domain	Strategy
Name the strategy	Conditional financial incentives
Define the strategy	A total of USD140 was administered as Visa cards for the completion of four lab visits and achievement of viral suppression measured twice during a 12-month period.
Specify the strategy	
(a) The Actors	Five diverse clinics including a non-profit health care center, federally qualified health center, hospital system, and two private practices participated in the study. Annual caseloads ranged from 200 to 1350. HIV clinic staff administered and tracked the distribution of incentives. One site had two locations. Another site had 8 staff members involved in the study to include in-house research staff to recruit, track, and report on participant progress. The remaining sites had 1–3 staff members dedicated to implementation.
(b) The Action	Confirmation of labs and viral load results, distribute financial incentive, record and document distribution of incentive, and report to the study team.
(c) Action Target	Adults living with HIV in the Baton Rouge area who are most likely to be established patients, patients who are not in HIV care, and patients who are at risk for falling out of HIV care.
(d) Temporality	Strategy occurred over a period of 12 months.
(e) Dose	Total of USD140 USD10 per lab (maxed at 4 labs) USD50 viral suppression at 6 months USD50 viral suppression at 12 months
Implementation outcomes affected	Clinical outcomes to include improvements in linkage to medical care, retention in medical care, and viral suppression.
Justification	Baton Rouge stakeholders prioritized this strategy to pilot within HIV clinics.

**Table 3:** BRPPS key implementation and RE-AIM (Reach, Effectiveness, Adoption, Implementation, Maintenance) constructs.

Variable/Construct	Measures	Sources	Methods	Measurement Period
Reach (penetration)				
a. Setting level	Number of enrolled participants among the estimated number of potentially eligible participants from the included clinics	HIV clinic sites	Surveys (*n* = 781) Clinical tracking forms	Baseline
Effectiveness				
a. Participant level	Engagement in HIV care Retention in HIV care Viral suppression	HIV clinic sites	EMR	12-months prior to enrollment, baseline, 6 and 12 months post enrollment
Adoption				
a. Setting level	Number of HIV clinic site that agreed to participate in the study. Number of HIV clinic sites that completed data collection activities and distributed financial incentives. Number of Incentives distributed	HIV clinic sites	Clinic incentive tracking forms	Ongoing
Implementation				
a. Acceptability	Level of agreement or disagreement with the extent to which financial incentives can improve overall health, improve viral load numbers, improve clinic attendance, and should not be provided to PLWH as it is something they should already be doing	PLWH	Surveys (*n* = 781)	Baseline
b. Feasibility	Ability to recruit enrollment targets (initially 1000 and then revised to 800 PLWH)	Site coordinators or delegates	Enrollment numbers	Years 1–2
c. Cost (cost, cost utility and cost threshold)	Client and implementation costs at each site as well as cost-effectiveness (i.e., new HIV infections averted)	PLWH HIV clinic sites	Surveys (*n* = 781) Micro-costing analysis worksheet	Baseline
d. Barriers	Barriers to implementation	HIV clinic staff	Calls with site coordinators	Ongoing calls
Maintenance (sustainability)				
a. Setting level	Resources (e.g., funding, staff)	HIV clinic sites	Calls with site coordinators	Ongoing calls

Legend: PLWH = Persons living with HIV; EMR = Electronic medical records.

**Table 4:** Baseline Demographic, Psychosocial, and Behavioral Characteristics of Study Participants enrolled in the BRPPS from four HIV clinics, Baton Rouge, LA (*n* = 781).

Characteristic (*n*)	*n* (%)
Age (*n* = 777)	
Mean (standard deviation)	42.4 (12.2)
Race (*n* = 769)	
Black/African American	686 (89.2)
White	63 (8.2)
Other	12 (1.6)
Multiracial	8 (1.0)
Ethnicity (*n* = 761)	
Hispanic/Latinx	12 (1.6)
Gender (*n* = 775)	
Male	412 (53.2)
Female	347 (44.8)
Transgender/non-binary	16 (2.1)
Sexual orientation (*n* = 738)	
Heterosexual or straight	500 (67.8)
Gay, lesbian, bisexual, or other same sex/fluid category	238 (32.2)
Relationship status (*n* = 777)	
Has primary partner	135 (17.4)
Education level (*n* = 748)	
Less than high school degree	245 (32.8)
High school degree, GED, or some college	398 (53.2)
Postsecondary degree (Associate’s, Bachelor’s, Master’s, or higher)	105 (14)
Employment status (*n* = 777)	
Unemployed	331 (42.6)
Employed full-time, part-time, or occasionally	290 (37.3)
Disabled	121 (15.6)
Other	35 (4.5)
Annual income (*n* = 642)	
Less than USD5000	273 (42.5)
USD5000–9999	99 (15.4)
USD10,000–14,999	76 (11.8)
USD15,000–19,999	45 (7.0)
USD20,000 or more	149 (23.2)
Housing status (*n* = 777)	
Has unstable housing	105 (13.5)
Incarceration history (*n* = 768)	
Recently incarcerated	90 (11.7)
Health Insurance (*n* = 756)	
Yes	678 (89.7)
Enrollment Category (*n* = 777)	
Category A eligible	451 (58)
Category B eligible	326 (42)
Internalized HIV Stigma (*n* = 717)	
Score (standard deviation)	4.6 (0.5)
Self-reported Medication Adherence (*n* = 619)	
Score (standard deviation)	11.8 (3.6)
Acceptability of Financial Incentives (*n* = 631)	
Mean (standard deviation)	2.5 (1.0)
Hazardous Alcohol Use (*n* = 684)	
Men (*n* = 370)	119 (32)
Women (*n* = 300)	88 (29)
Transgender individuals (*n* = 14)	1 (7)
Depressive Symptoms (*n* = 661)	
Score (standard deviation)	7.2 (7.1)
Years living with HIV (*n* = 727)	
Less than 1 year	62 (8.5)
1 to 5 years	178 (24)
More than 5 years	487 (67)
HIV care outcomes at enrollment (*n* = 781)	
Engaged in care	548 (70)
Retained in care	326 (42)
Virally suppressed	485 (62)

**Table 5:** Baseline correlates of HIV continuum of care outcomes at 12-month follow-up, BRPPS, Baton Rouge, LA *.

Engaged in Care (*n* = 735)			Retained in Care (*n* = 735)		Virally Suppressed (*n* = 735)	
Variables	Coef. (SE)	*p*	Coef. (SE)	*p*	Coef. (SE)	*p*
Baseline HIV care outcomes	1.32 (0.33)	0.000	0.44 (0.19)	0.023	0.33 (0.18)	0.062
Enrollment Site (reference = Site A)						
Site B	–0.40 (0.51)	0.434	–0.25 (0.27)	0.356	–0.65 (0.24)	0.007
Site C	–0.43 (0.45)	0.339	–0.39 (0.22)	0.080	–0.34 (0.18)	0.064
Site D **	–1.84 (0.72)	0.011	–0.87 (0.50)	0.080	–	–
Age at entry	0.02 (0.01)	0.082	0.03 (0.01)	0.001	0.02 (0.01)	0.029
Race (reference = White)						
Black	0.46 (0.66)	0.481	0.24 (0.35)	0.495	–0.20 (0.30)	0.497
Other race	0.09 (1.36)	0.950	0.76 (0.84)	0.364	0.90 (0.74)	0.226
Multiracial	–0.20 (1.08)	0.851	–0.05 (0.78)	0.948	–0.10 (0.83)	0.901
Hispanic/Latinx	–0.34 (1.29)	0.793	–0.03 (0.75)	0.972	–1.43 (0.89)	0.110
Gender (reference = Female)						
Male	–0.38 (0.34)	0.257	0.06 (0.18)	0.742	–0.10 (0.16)	0.548
Transgender or non-binary	–0.82 (0.69)	0.233	1.20 (0.72)	0.094	0.81 (0.52)	0.117
Has primary partner	–0.20 (0.41)	0.618	0.11 (0.23)	0.624	0.03 (0.21)	0.903
Unemployed	–0.26 (0.30)	0.391	–0.01 (0.18)	0.944	0.07 (0.16)	0.651
Unstable housing	–0.73 (0.37)	0.051	–0.24 (0.26)	0.359	–0.17 (0.23)	0.464
Has health insurance	0.43 (0.40)	0.282	–0.02 (0.29)	0.945	0.00 (0.27)	0.991
Enrollment category (reference = category B)						
Category A eligible	0.55 (0.38)	0.144	0.39 (0.20)	0.053	0.50 (0.18)	0.005
Heterosexual or Straight Income (reference = USD20,000 or more)	0.09 (0.41)	0.818	0.45 (0.24)	0.057	0.11 (0.21)	0.617
USD0–USD9000	–0.19 (0.48)	0.696	0.02 (0.26)	0.949	–0.18 (0.24)	0.457
USD10,000–USD19,000	1.40 (0.90)	0.121	0.33 (0.31)	0.284	0.04 (0.27)	0.879
Highest education (reference = postsecondary degree)						
Less than high school degree	0.24 (0.51)	0.645	0.10 (0.30)	0.740	0.08 (0.27)	0.764
High School degree, GED, or some college	0.30 (0.44)	0.501	0.24 (0.27)	0.384	0.00 (0.25)	0.984
Years living with HIV (reference = 5 or more years)						
Less than a year	–0.23 (0.65)	0.716	0.81 (0.41)	0.049	0.81 (0.32)	0.011
1–4 years	–0.39 (0.35)	0.264	–0.20 (0.21)	0.343	–0.13 (0.21)	0.514
Ever incarcerated	–0.96 (0.38)	0.012	–0.19 (0.27)	0.479	–0.44 (0.27)	0.104
Internalized HIV Stigma	–0.02 (0.34)	0.962	–0.29 (0.18)	0.103	0.10 (0.15)	0.501
Self-reported Medication Adherence	0.04 (0.05)	0.428	0.00 (0.03)	0.886	0.08 (0.03)	0.002
Depressive Symptoms	0.06 (0.03)	0.051	0.01 (0.01)	0.520	0.01 (0.01)	0.283
Alcohol Use Index	–0.06 (0.06)	0.290	0.00 (0.04)	0.961	–0.04 (0.03)	0.228
Acceptability of financial incentives	0.03 (0.16)	0.850	–0.09 (0.10)	0.332	–0.16 (0.09)	0.071

* The benchmark model incorporates a limited set of covariates that had relatively complete responses in order to maximize the number of participants included in the analytic samples (*n* = 735). Sample sizes for the models that included additional covariates (those listed below the benchmark model) varied due to a higher degree of missing or incomplete responses on the baseline questionnaire. The coefficients presented represent the regression estimate for each additional covariate when it is added to the benchmark model. The sample sizes are as follows: Heterosexual or straight (*n* = 707), Income (*n* = 608), Highest education (*n* = 714), Years living with HIV (*n* = 698), Ever incarcerated (*n* = 729), Internalized HIV Stigma (*n* = 674), Self-reported Medication Adherence (*n* = 587), Depression Symptoms (*n* = 638), Alcohol Use Index (*n* = 645), and Acceptability of financial incentives (*n* = 604). ** No participants were virally suppressed at this site at 12-month follow-up.

**Table 6:** Cost utility analysis results from three HIV clinics, BRPPS, Baton Rouge, LA.

	Clinic A	Clinic B	Clinic C
C			
Implementation cost over a one- year period	USD396,910.00	USD17,198.05	USD24,341.04
T			
Treatment cost of one HIV infection from literature (2019)	USD382,954	USD382,954	USD382,954
A			
Net number of individuals virally suppressed during study	22	6	9
Number of infections averted	1.34	0.37	0.55
Q			
QALYs saved through improved individual health (Q1 or 0.039 * NetA)	0.86	0.23	0.35
QALYs saved through averted HIV infection (Q2 or 5.83 * A)	7.81	2.16	3.21
Total QALYs saved (Q1 + Q2)	8.67	2.39	3.56
Cost Utility Ratio	USD10,006.06	USD140,783.59	USD95,139.77

**Table 7:** Cost threshold analysis results from three HIV clinics, BRPPS, Baton Rouge, LA.

	Clinic A	Clinic B	Clinic C
Number of Clients enrolled	214	106	227
Client-related costs	USD15,515.00	USD1303.80	USD822.88
Staff-related costs	USD164,781.00	USD5819.24	USD18,287.84
Materials/other consumables	USD216,614.00	USD8499.90	-
Total cost—**societal perspective**	USD396,910.00	USD17,198.05	USD24,341.04
Expressed per client	USD1854.72	USD162.25	USD107.23
Expressed per contact	USD618.24	USD24.57	USD53.61
Total cost—**payor’s perspective**	USD381,395.00	USD15,894.25	USD23,518.16
Expressed per client	USD1782.22	USD149.95	USD103.60
Expressed per contact	USD594.07	USD22.71	USD51.80
Cost-Saving Threshold: Number of HIV transmissions to be averted (C/T)	1.04	0.04	0.06
Cost-Effective threshold: Number of QALYs to be saved (C/W)	2.03	0.09	0.12

## Data Availability

Due to the nature of this research, participants did not provide consent for their data to be made publicly available to other researchers, so supporting data are not available.
